# The SYSCID map: a graphical and computational resource of molecular mechanisms across rheumatoid arthritis, systemic lupus erythematosus and inflammatory bowel disease

**DOI:** 10.3389/fimmu.2023.1257321

**Published:** 2023-11-01

**Authors:** Marcio Luis Acencio, Marek Ostaszewski, Alexander Mazein, Philip Rosenstiel, Konrad Aden, Neha Mishra, Vibeke Andersen, Prodromos Sidiropoulos, Aggelos Banos, Anastasia Filia, Souad Rahmouni, Axel Finckh, Wei Gu, Reinhard Schneider, Venkata Satagopam

**Affiliations:** ^1^ Luxembourg Centre for Systems Biomedicine, University of Luxembourg, Esch-sur-Alzette, Luxembourg; ^2^ ELIXIR Luxembourg, Esch-sur-Alzette, Luxembourg; ^3^ Institute of Clinical Molecular Biology, Christian-Albrechts-University Kiel and University Medical Center Schleswig-Holstein, Kiel, Germany; ^4^ Department of Internal Medicine I, University Medical Center Schleswig-Holstein, Kiel, Germany; ^5^ Diagnostics and Clinical Research Unit, Institute of Regional Health Research, University Hospital of Southern Denmark, Aabenraa, Denmark; ^6^ Institute of Molecular Medicine, University of Southern Denmark, Odense, Denmark; ^7^ Rheumatology and Clinical Immunology, Medical School, University of Crete, Heraklion, Greece; ^8^ Laboratory of Rheumatology, Autoimmunity and Inflammation, Institute of Molecular Biology and Biotechnology, Foundation for Research and Technology (IMBB-FORTH), Heraklion, Greece; ^9^ Autoimmunity and Inflammation Laboratory, Biomedical Research Foundation of the Academy of Athens, Athens and Laboratory of Molecular Hematology, Democritus University of Thrace, University Hospital of Alexandroupolis, Alexandroupolis, Greece; ^10^ Unit of Animal Genomics, GIGA-Institute, University of Liège, Liège, Belgium; ^11^ Rheumatology Division, Geneva University Hospital (HUG), Geneva, Switzerland; ^12^ Geneva Center for Inflammation Research (GCIR), University of Geneva (UNIGE), Geneva, Switzerland

**Keywords:** inflammatory bowel disease, rheumatoid arthritis, systemic lupus erythematosus, molecular mechanisms, curation, pathway biology, systems biology

## Abstract

Chronic inflammatory diseases (CIDs), including inflammatory bowel disease (IBD), rheumatoid arthritis (RA) and systemic lupus erythematosus (SLE) are thought to emerge from an impaired complex network of inter- and intracellular biochemical interactions among several proteins and small chemical compounds under strong influence of genetic and environmental factors. CIDs are characterised by shared and disease-specific processes, which is reflected by partially overlapping genetic risk maps and pathogenic cells (e.g., T cells). Their pathogenesis involves a plethora of intracellular pathways. The translation of the research findings on CIDs molecular mechanisms into effective treatments is challenging and may explain the low remission rates despite modern targeted therapies. Modelling CID-related causal interactions as networks allows us to tackle the complexity at a systems level and improve our understanding of the interplay of key pathways. Here we report the construction, description, and initial applications of the SYSCID map (https://syscid.elixir-luxembourg.org/), a mechanistic causal interaction network covering the molecular crosstalk between IBD, RA and SLE. We demonstrate that the map serves as an interactive, graphical review of IBD, RA and SLE molecular mechanisms, and helps to understand the complexity of omics data. Examples of such application are illustrated using transcriptome data from time-series gene expression profiles following anti-TNF treatment and data from genome-wide associations studies that enable us to suggest potential effects to altered pathways and propose possible mechanistic biomarkers of treatment response.

## 1 Introduction

Chronic inflammatory diseases (CIDs) are a group of incurable immune system disorders of unclear aetiology characterised by a persistent inflammation that ultimately cause damage in target tissues and organs (1). Currently, there are no curative treatment options for CIDs. Available targeted treatments are only beneficial in a subset of patients. Despite this heterogeneity of response, currently no biomarkers are available, which would allow stratifying patients into different therapeutic modalities. Even the most advanced treatment options have one severe limitation: they only offer short drug-free remission periods and therefore have to be given lifelong (2). Regarding prevalence, CIDs, more specifically their subgroup of immune-mediated inflammatory diseases, are estimated to affect 5 to 7% of the Western population (3). Finally, CIDs can result in debilitating physical and psychosocial symptoms for patients and are a sizable burden to society through loss of education, absenteeism, and increasing health-care costs (4, 5).

Several molecular mechanisms underlying CIDs have been converted into clinical treatments (2, 6). For example, biologics, mainly monoclonal antibodies targeting specific cytokines, emerged as clinical treatment options; however, their therapeutic potential is not always achieved in clinical practice (7). This may be explained by a switch towards alternative molecular pathways that are insensitive to the mechanism of action of the biologic (8–11).

Finding such alternative molecular pathways is thus important for understanding the molecular mechanisms of the disease and for indicating new potential targets for complementary biologics to achieve better clinical responses. In this regard, the results of clinical trials of so-called dual targeted therapy for CIDs showed promising results (12, 13). As CIDs emerge from a complex network of causal interactions between proteins, small chemical compounds, environmental factors and different types of cells, the identification of such alternative pathways and their targets should be based on their role in the network to assure their downstream effects. Such exercise requires, however, the construction and the analysis of a network encompassing CID-related causal interactions – events in which a phenotype or state of one biomolecule is affected by another phenotype or biomolecule under the context of the CID of interest –, covering relevant mechanistic details.

In response to this demand, we present the SYSCID map (https://syscid.elixir-luxembourg.org/), a network of mechanistically resolved causal interactions related to three CIDs - inflammatory bowel disease (IBD), rheumatoid arthritis (RA) and systemic lupus erythematosus (SLE) (https://syscid.elixir-luxembourg.org). As these CIDs share a significant amount of genetic disease risk loci and immunological features as well as large data sets and clinically validated markers of individual disease phenotypes (1), we consider them an important starting point for systematic exploration of common and specific mechanisms involved in the pathophysiology of CIDs. The SYSCID map is constructed based on literature review and represents disease mechanisms as an interactive diagram following graphical systems biology standards. The SYSCID map is a standardised knowledge repository for the IBD, RA and SLE and a hypothesis-generating resource for new discoveries. Here, we demonstrate how the SYSCID map can be used for these purposes. First, we discuss the scope of the literature used to construct the map as an interactive, graphically guided review of IBD, RA and SLE molecular pathogenesis. We then demonstrate the process of omics data interpretation using the map to identify potential effects of altered pathways and propose mechanistic biomarkers (14).

## 2 Methods

### 2.1 Map construction and availability

**Figure 1:**
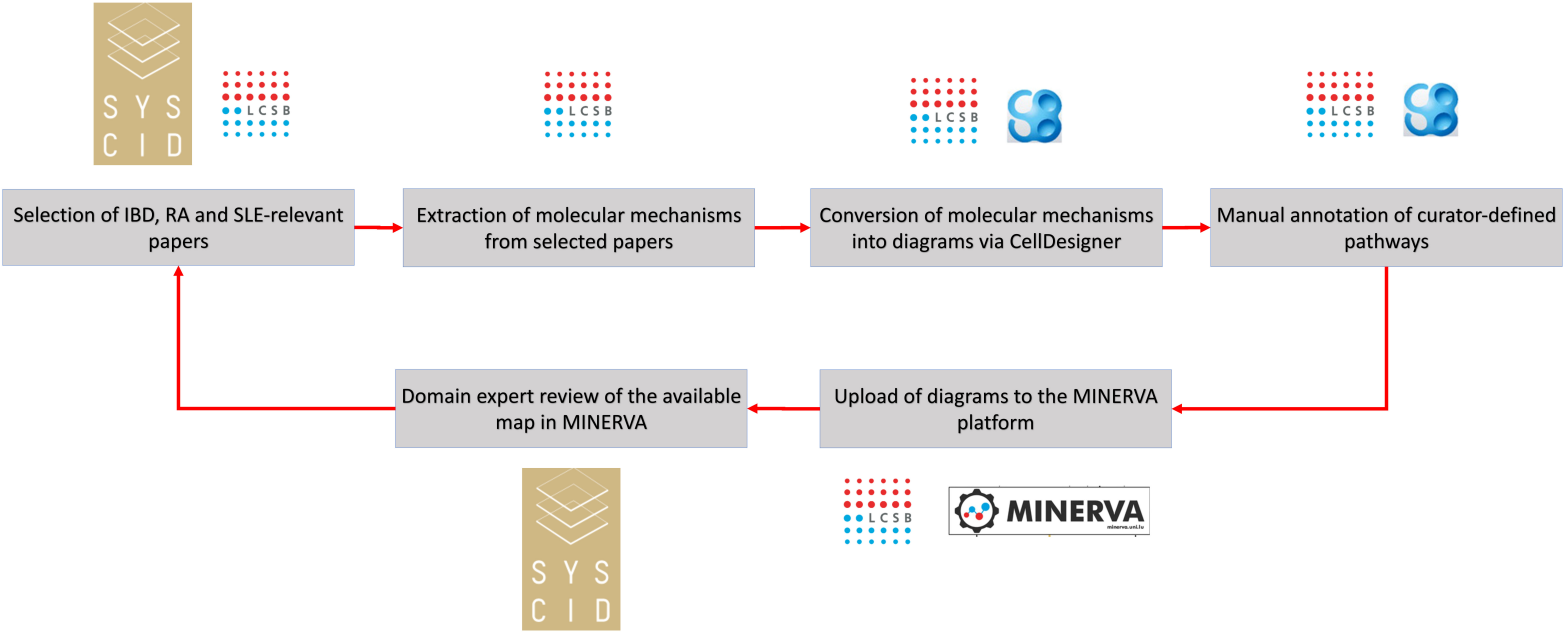
Building the SYSCID map Domain experts from the SYSCID project selected and provided curators with the papers potentially reporting molecular processes related to inflammatory bowel diseases (IBD), rheumatoid arthritis (RA) and systemic lupus erythematosus (SLE). Curators then extracted relevant molecular processes from the selected papers and encoded the extracted knowledge into the CellDesigner format; in addition, curators manually defined pathways, i.e., subsets of molecular processes leading to a specific phenotype, covering specific regions of the map. The SYSCID map was uploaded to the MINERVA platform and then made publicly accessible so that SYSCID domains experts could review it. After some rounds of reviews and refinement, a final improved version of the map was uploaded to MINERVA.

Knowledge about IBD-, RA- and SLE-relevant causal interaction were captured from biomedical literature and then encoded into a diagrammatic visualisation format, i.e., the map, by using the CellDesigner tool (15) following the Systems Biology Graphical Notation (SBGN) standard (16; https://sbgn.github.io/learning); the only exception is the representation of drugs for which we used the CellDesigner own glyph. The SYSCID map was then uploaded into the MINERVA platform (17) (https://syscid.elixir-luxembourg.org/minerva/) for easy access and exploration (**Figure 1**). Details are found below.

### 2.2 Capturing IBD-, RA- and SLE-related causal interactions from literature

The SYSCID map was constructed by following most of the recommendations in the recently published guidelines for disease maps construction (18). At first, we manually curated reviews and original peer-reviewed research papers suggested by domain experts from the SYSCID consortium. These papers were published between 1989 and 2021, being most of them (~88%) published between 2010 and 2020 (see **Table S10** for the list of Pubmed IDs of curated papers). While most of these domain expert-selected papers describe relevant experimentally proven causal interactions under the context of IBD, RA and SLE, some other papers describe experimentally proven general immune processes-related causal interactions that were added to the map to cover missing disease-specific information. Most of causal interactions were directly assigned to compartments (i.e., organs, tissues, cells and organelles) based on information present in the paper itself; in some cases, this assignment was inferred based on common knowledge about the most usual locations where a given causal interaction takes place. We also captured details concerning causal interactions (e.g., gene variant and phosphorylated or citrullinated residues) when they were available in the paper.

### 2.3 Encoding causal interactions into diagrams

The retrieved causal interactions were then encoded into a diagrammatic visualisation format using the CellDesigner tool (15) by adopting its own standard visual syntax, the CellDesigner’s Systems Biology Markup Language (SBML) extension. The encoding process was performed following the Systems Biology Graphical Notation (SBGN) standard (16; https://sbgn.github.io/learning). We used both SBGN Process Description (PD) and Activity Flow (AF) languages. In PD, a causal interaction is represented as a state transition of a biochemical entity (regulated entity) with biochemical details about the type of molecular transition and how it is modified by a regulator entity. In AF, a causal interaction is represented as a simple directed connection between two entities with information about the type of regulation, i.e., activation or inhibition.

### 2.4 Identification and annotation of the map elements

All entities (proteins, RNAs, genes, complexes, metabolites, drugs and phenotypes), compartments (organelles, cells, tissues and organs) and interactions in the map were properly annotated to ensure the map interoperability with systems biology tools and external databases. For this end, we followed the Minimal Information Requested In the Annotation of Models (MIRIAM) (19), a standard for annotating and curating computational models and maps. Proteins, RNAs and genes were identified by their corresponding HUGO Gene Nomenclature Committee (HGNC, https://www.genenames.org) official symbols so that the MINERVA platform, the tool used to for visualization and navigation through the map (see more details below), could automatically assign additional annotations (Ensembl, Entrez Gene, RefSeq and Uniprot identifiers [IDs]) to these entities; in case of symbols or terms not recognized by HGNC, these additional annotations were manually assigned. Complexes were identified by their Gene Ontology (GO) cellular component terms and corresponding IDs (20, 21). Metabolites were identified by their ChEBI names (22; https://www.ebi.ac.uk/chebi) and corresponding IDs. Drugs were identified by their Drugbank (23); https://go.drugbank.com/) generic names and corresponding IDs. Phenotypes were identified by their GO biological process terms and corresponding IDs (http://geneontology.org/docs/ontology-documentation) (20, 21) in case of phenotypes representing biological processes or Medical Subject Headings (MeSH) terms and corresponding IDs (https://www.ncbi.nlm.nih.gov/mesh) in case of phenotypes representing disease-related elements.

For annotations not automatically retrieved by MINERVA, we used the MIRIAM-dedicated section of CellDesigner to add annotations. For adding references to the interactions, i.e., Pubmed IDs of the papers from which the interactions were collected, we used the relation “bqbiol: isDescribedby”; for adding all other annotations, we used the relation “bqmodel:isEncodeby”.

### 2.5 Map availability and visualisation in the MINERVA platform

The SYSCID map is available as an online interactive map using the Molecular Interaction NEtwoRks VisuAlization (MINERVA) platform (17) (https://syscid.elixir-luxembourg.org/minerva/). MINERVA is a standalone web server for visual exploration, analysis and management of molecular networks encoded in systems biology formats, including CellDesigner, SBML and SBGN. MINERVA provides automated content annotation and verification and enables, among other features, the overlaying of experimental data (e.g., transcriptomics, gene variant data etc) on the visualised networks. For more details about the functionalities available in MINERVA, please consult the MINERVA documentation at https://minerva.pages.uni.lu/doc/.

### 2.6 Integration of omics data to the map

IBD-, RA- and SLE-associated variants extracted from the Gene Wide-Association Studies (GWAS) Catalogue (24) and SYSCID-derived transcriptome data were integrated to the SYSCID map by using the MINERVA platform as detailed described below and following the instructions provided in the MINERVA pages (https://minerva.pages.uni.lu/doc/user_manual/v16.0/index/#upload-of-user-provided-overlay-data).

#### 2.6.1 Integration of GWAS catalogue data to the map

For the analysis of possible mechanistic effects of IBD, RA and SLE-associated gene variants on downstream molecular processes in SYSCID map, we first obtained relevant data from the Gene Wide-Association Studies (GWAS) catalogue (24). We collected data on 04.11.2022 by using the Experimental Factor Ontology (EFO) identifiers of IBD (EFO_0003767 for IBD, EFO_0000384 for Crohn’s disease and EFO_0000729 for ulcerative colitis), RA (EFO_0000685) and SLE (EFO_0002690).

We filtered each of the three datasets - IBD, RA and SLE - to exclude variants that are intergenic or fall into non-coding transcripts (mature miRNA, non-coding transcript exon, non-coding transcript intron and non-coding transcript splice region). We excluded intergenic variants as they do not seem to have a relationship with the closest genes as indicated in GWAS but, instead, they seem to impact more distant genes, even those located in different chromosomes (25). Variants in non-coding transcripts are irrelevant to the SYSCID map as virtually all causal interactions in the map take place between proteins or DNA binding transcription factors and their protein-coding target genes. Finally, we prepared a MINERVA-compatible file for the creation of a visual overlay of these variants in SYSCID map in the MINERVA platform (https://syscid.elixir-luxembourg.org/, “General Overlays” tab).

#### 2.6.2 Integration of transcriptome data to the map

For the analysis of transcriptome data integration with the map, we used the transcriptome dataset published by Mishra et al. (26) concerning the detection of DEGs by comparing gene expression profiles of RNA extracted from whole blood of remitting and non-remitting IBD patients at six time points (4, 24 and 72 h and 2, 6 and 14 weeks) after infliximab exposure with gene expression profiles determined in baseline, i.e., before drug exposure. We focused specifically on DEGs determined at 4 h in remitting patients as, according to authors, the most profound alterations occurred at this time point. This dataset is available at the NCBI GEO website under the accession number GSE191328 (https://www.ncbi.nlm.nih.gov/geo/query/acc.cgi?acc=GSE191328 and via the Supplementary Information of the original paper.

We downloaded the dataset from the above-mentioned **Supplementary Information** and extracted the columns related to 4 h after drug exposure, i.e., LFC_4h and padj_4h, and the column containing DEG’s HGNC symbols. We removed DEGs with an adjusted p-value ≥ 0.05 (in column padj_4h) and then normalized the log2 fold change values (in column LFC_4h) of remaining DEGs to the [-1,1] range (**Table S6**). Finally, we prepared the file for the creation of a visual overlay of these DEGs in SYSCID map in the MINERVA platform (https://syscid.elixir-luxembourg.org, “General overlays” tab).

#### 2.6.3 Selection of proteins matched against variants and DEGs for further analysis

After integrating variant data from the GWAS catalogue and selected DEGs from Mishra et al. (26) in SYSCID map, we shortlisted the proteins matched against variants and DEGs, i.e., possibly altered proteins, for potential further analyses. To this end, possibly altered proteins were eligible for potential further analyses if they were (1) activating, inhibiting or catalysing at least one downstream molecular process or (2) activating or inhibiting a downstream molecule. Among the eligible proteins, we prioritized pairs of possibly altered proteins directly linked by a molecular process.

#### 2.6.4 Functional enrichment analysis in MINERVA

To check if some of the manually annotated pathways in the SYSCID map were enriched in IBD, RA or SLE-associated gene variants or DEGs, we used the MINERVA’s GSEA plugin (27) as described in detail here: https://minerva.pages.uni.lu/doc/plugins/gsea-plugin/. In brief, the MINERVA’s GSEA plugin considers as the background gene list all genes present in the map; as the pathway database source, the plugin considers the list of annotated pathways (**Table S1**) in the own map. The statistical test used is the hypergeometric test and the obtained p-values are adjusted for multiple comparisons by using the Bonferroni test. Enriched pathways were those with adjusted p-value < 0.05.

## 3 Results

### 3.1 Aim and structure of the SYSCID map

**Figure 2:**
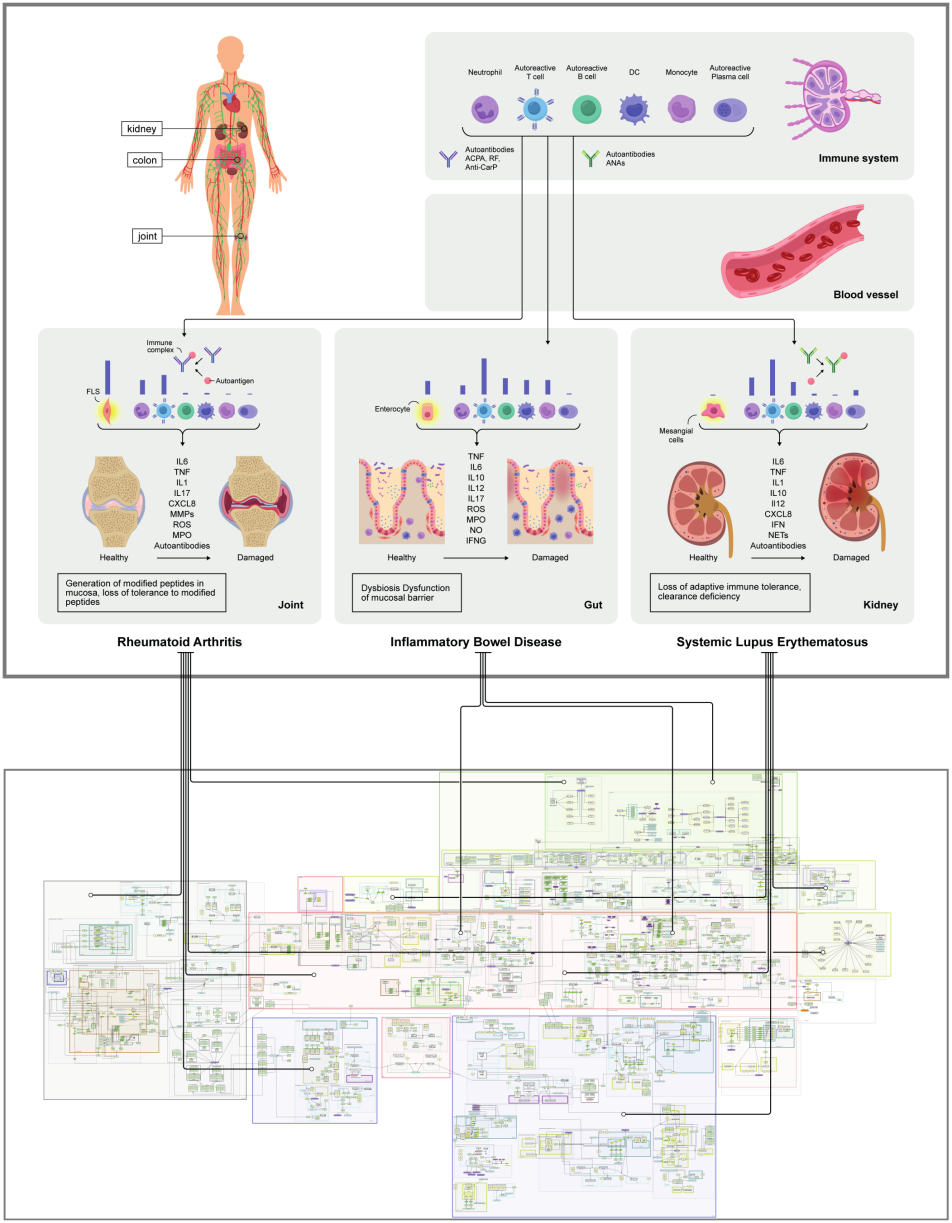
General overview of the SYSCID map The upper panel shows a pictorial overview of the most relevant IBD, RA and SLE-related organs, tissues, phenotypes, cells and molecules; the lower panel shows the MINERVA network visualization of the SYSCID map (https://syscid.elixir-luxembourg.org) with all its molecular entities and causal interactions confined in different compartments, e.g., cells and organs. The disease-specific causal interactions tend to occur in certain niches in the map; for instance, while RA-related interactions tend to happen in synovial joint, lymphoid organs, blood vessel, gut lumen and oral cavity, SLE-related interactions tend to take place in lymphoid organs, blood vessel, kidney and skin; IBD-related interactions, in turn, tend to appear mainly in gut (lumen, epithelium and lamina propria) and blood vessels.

CIDs, such as IBD, RA and SLE, are characterised by a chronic inflammatory state that emerges from a complex network of inter- and intracellular biochemical interactions among several proteins and small chemical compounds under strong influence of genetic and environmental factors (1). The SYSCID map focuses on presenting this complex network as a diagrammatic visualisation using systems biology standards (**Figure 2**). By using the MINERVA Platform (17, 27), users can explore the map interactively, request specific knowledge and visualise omics data contextualising it to related disease mechanisms. Contents of the SYSCID Map are annotated using ontologies and controlled vocabularies, and available to connect to computational workflows via a programmatic endpoint. The SYSCID map can be freely accessible by accessing https://syscid.elixir-luxembourg.org.

**Figure 3:**
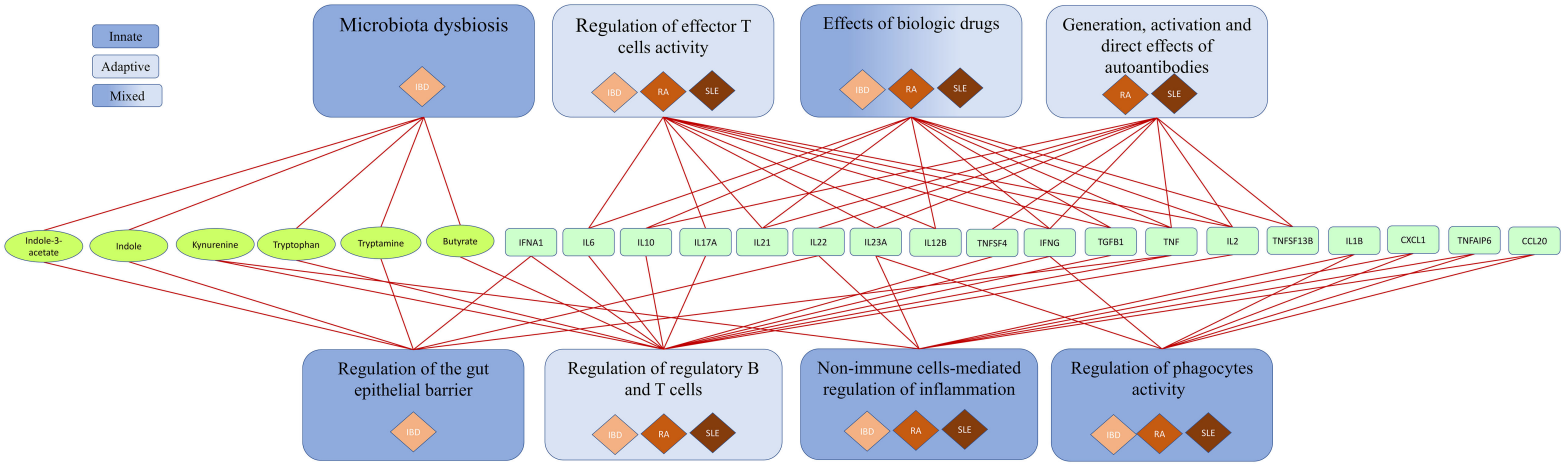
Pathophysiological features of IBD, RA and SLE in SYSCID map The 66 pathways in the map can be grouped into eight major pathophysiological features - associated with adaptive, innate or mixed immune system - and are linked to each other via six bacterial or endogenous metabolites (ellipses), 17 cytokines and a cytokine-induced protein (TNFAIP6) (rectangles containing official gene symbols from the Human Gene Nomenclature Committee [HGNC]).

The SYSCID map contains 1494 causal interactions between 714 molecules (341 proteins, 173 mRNAs, 76 genes, 124 complexes, 31 metabolites and 20 drugs), 80 different cell types (taking into consideration general types and different states of the same cell type, e.g., leucocyte and mature and immature plasmacytoid dendritic cells) and 131 phenotypes (24 pathological and 107 non-pathological processes) that are distributed across compartments representing organs and tissues relevant for each disease: gut (lamina propria, epithelium and lumen), skin, oral cavity, adipose tissue, kidney, synovial joint, primary (thymus) and secondary (lymph node, spleen and bone marrow) lymphatic organs and peripheral blood (**Figure 2**). Molecular mechanisms of selected biologics used to treat CIDs are depicted in the submap “Anti-inflammatory mechanisms of selected biologics” embedded in the main map (**Table S1**). Additionally, to facilitate both exploration of molecular processes and visualisation of omics data, we manually annotated 66 pathways (**Table S1**) - subsets of molecular processes linked to a specific phenotype or biologic - covering most of the map. These pathways were grouped into eight major pathophysiological features of CIDs as shown in **Figure 3** and **Table S1**.

### 3.2 The SYSCID map contents: a graphical review of mechanisms at the crosstalk of IBD, RA and SLE

The SYSCID map was built based on the review of IBD-, RA- and SLE-relevant literature and, as such, the exploration of SYSCID map contents leads naturally to a graphical review of key molecular mechanisms of these diseases. In this section, we summarize the contents of the map and try to show that it can serve the purpose of a user-friendly graphical review. To this end, we grouped the above-mentioned 66 manually annotated pathways into eight main pathophysiological features of these CIDs - that were, in turn, grouped into three types of immune system responses - and briefly described some relevant aspects of each pathway (**Figure 3** and **Table S1**).

#### 3.2.1 Innate immune system

##### 3.2.1.1 Non-immune cells-mediated regulation of inflammation

The immune system is regulated not only by immune cells, but also by non-immune cells, such as epithelial cells, epidermal keratinocytes and synoviocytes, that, beyond forming a physical barrier against external agents, also produce bioactive effectors and regulators of the immune response (28). Therefore, it is expected that abnormal activities of non-immune cells related to the immune system regulation may be involved in the pathogenesis of inflammatory diseases (28). The SYSCID map depicts some of the molecular processes involved in these abnormal activities regarding fibroblast-like synoviocytes (FLS), gut epithelial cells and epidermal keratinocytes (**Figure 3**). FLSs, the dominant non-immune cells of synovial tissues, contribute to joint inflammation and destruction in RA (29). The SYSCID map illustrates regulation of production of molecules promoting joint destruction by FLS, including alarmins (30) and adipokines. (31, 32). Alarmins (S100A8 and S100A9), stimulated by IL22, and MMP1, MMP3 and prostaglandin E2, induced by adipokine, are all associated to joint destruction (33–35). Another FLS-driven proinflammatory mechanism is production of CXCL2, CXCL8, IL6 and PTGS2, regulated by the RNA-binding protein ZFP36 (36) and the proinflammatory interleukin IL1B. In addition to IL1B, the FLS activity is also stimulated by TNF. The SYSCID map illustrates regulation of TNF-induced inflammatory programs by MTORC1 depending on the availability of amino acids glutamine, arginine and leucine (37). The presence of these amino acids favours the TNF induction of STAT1-dependent genes, such as *CXCL11* and *TNFSF13B*, while their absence leads to TNF-induced expression of NFkB-dependent genes, such as *IL6, CXCL8* and *PTGS2* (37). As shown in the map, gut epithelial cells can also modulate the immune response via the production of proinflammatory proteins mediated by microbial tryptophan-derived metabolites (38), endoplasmic reticulum stress (39) and gasdermin-D (40).

##### 3.2.1.2 Regulation of phagocytes activity

Macrophages and neutrophils are key regulators of inflammatory processes via the production of cytokines and chemokines, extracellular traps (ETs) or through the interaction with other immune cells (41, 42). Their activities are tightly regulated, and their dysfunction is associated with the three CIDs as illustrated in the SYSCID map (**Figure 3**). One of encoded mechanisms is CEBPD-mediated regulation of macrophage activity in RA (43), representing pro-inflammatory protein IL1B promoting the expression of the transcription factor CEBPD, which regulates several pro-inflammatory proteins. Another mechanism of regulation involves TLR3-induced PKM (44) linking glycolysis pathway and pro-inflammatory activity of macrophages in RA (Vander 45). Still, the SYSCID map encodes a novel function for pro-apoptotic protein BCL2L11 in SLE (46); this protein suppresses pro-inflammatory macrophage activity by inhibiting TBK1 kinase that, in turn, activates IRF3, a transcription factor that promotes the expression of several pro-inflammatory proteins (47). Finally, regarding regulation of neutrophils activity, the SYSCID map shows that ETs, which promote IBDs through the impairment of gut epithelial barrier (48), are generated by PADI4-catalyzed citrullination of H3 histones in neutrophils (49).

##### 3.2.1.3 Regulation of the gut epithelial barrier

The intestinal epithelial barrier (IEB) is one of the largest interfaces between the external and body environments (50). The IEB is tightly regulated, and its pathology may contribute to the aetiology of IBD, but - via licensing of immune responses - also to other CIDs such as RA and SLE (51). Intercellular junction structures (52) and the activities of gut microbiota and intraepithelial lymphocytes (IELs) are pivotal regulators of IEB (53). Molecular mechanisms involved in these three types of IEB regulation are present in the SYSCID map. One of them is regulation of the stability of gut epithelial adherens junctions involving INAVA, E-cadherin and CYTH1-activated ARF6 (54, 55). The map shows how the absence of anti-inflammatory immune cells (e.g., CD4/CD8αα T cells) as well as the context-dependent pro- and anti-inflammatory effects of microbiota-derived tryptophan metabolites (e.g., indole, indole-3-acetate, tryptamine) and cytokines (e.g., IL22) are important for the IEB homeostasis. Concerning IL22, the SYSCID map illustrates pro- and anti-inflammatory outcomes regulated by this interleukin, and its regulation by the protein ATG16L1 in engaging the CGAS–STING pathway (56).

##### 3.2.1.4 Microbiota dysbiosis

Microbiota dysbiosis is a disruption to the microbiota homeostasis in tissues in direct contact with the external environment caused especially in response to exposure to environmental chemicals and food components. Resulting changes in the microbiota composition are linked to IBD, RA and SLE (57–59). Microbiota-derived metabolites are particularly implicated in the pathogenesis of CIDs as they seem to be essential in maintaining immunological equilibrium (60). Moreover, microbial mimic peptides are increasingly associated with CIDs; in this case, similarities between foreign and self-peptides favour an activation of autoreactive immune cells (61). The SYSCID map shows which bacterial genera produce immunoregulatory small compounds in IBD, e.g., *Roseburia* and *Faecalibacterium* for butyrate and *Bifidobacteirum* for acetate, and mimic peptides in RA, i.e., *Eggerthella, Clostridium, Bacteroides, Cryptobacterium, Actinomyces, Atopobium* and *Oribacterium* for peptides like COL11A2 and HLA-DRB1*0401. Moreover, the SYSCID map also displays which metabolic processes are altered due to microbiota dysbiosis in the CIDs of interest, such as the glucuronate breakdown in IBD, and the heme metabolic process in RA.

#### 3.2.2 Adaptive immune system

##### 3.2.2.1 Generation, activation and direct effects of autoantibodies

Self-reactive cytotoxic and helper T cells and self-reactive B cells with their terminally differentiated counterparts, the antibody-secreting plasma cells, are hallmarks of SLE and RA. Due to the failure of the peripheral tolerance processes, self-reactive naive T and B cells are not eliminated and become activated instead (62). RA- and SLE-relevant mechanisms associated with differentiation of self-reactive B and plasma cells are represented in SYSCID map such as, for example, the mechanisms of B cells differentiation stimulated by IL10, secreted either by dendritic cells (63) or by Th1 cells (64). Dendritic cells also influence B cell differentiation via TLR-activated signalling protein MYD88 (65). Moreover, the SYSCID map illustrates B cell differentiation stimulated by IL4 produced by Tfh2 cells, and its modulation by transcription factor ETS1 (66). Finally, B cell differentiation can also take place outside germinal centres driven by TLR7 in an IL21-mediated fashion (67–69).

The abovementioned activation of self-reactive plasma cells drives the production and activation of autoantibodies, a hallmark of RA and SLE. In RA, the most common and specific autoantibodies are against citrullinated protein antigens (ACPAs), mostly derived from proteins that are highly expressed but not specific for synovial joints such as vimentin and fibrinogen (70, 71). The SYSCID map depicts the presence of some of these ACPAs (72, 73) in joint and secondary lymphoid organs, and their general (arthritis) and specific (osteoclastogenesis) effects. (72, 74–76). The SYSCID map also illustrates the association of glycosylation level of antibodies with their level of inflammatory activity (77), particularly the effect of the IL23A-Th17 cell-IL22 axis on the inhibition of the expression of the ST6GAL1 enzyme in plasmablasts, driving secretion of desialylated autoantibodies with a proinflammatory activity (78).

##### 3.2.2.2 Regulation of effector T cells activity

Effector helper (CD4) and cytotoxic (CD8) T cells play a central role in regulation of the immune system, and their regulation is closely related to IBD (79–81), RA (82) and SLE (83, 84). While helper T cells produce and release cytokines to aid other immune cells, cytotoxic T cells directly kill cells expressing antigens on their surface. The SYSCID map contains several pathways describing the activity of effector T cells and how these cells are regulated at multiple levels. At the innate immune system level, the map shows that T cells are indirectly regulated by Toll-like receptors (TLRs) pathways in conventional dendritic cells via the adaptor protein MYD88 in SLE (65). At the adaptive immune system level, the SYSCID map shows that T cells can be regulated via the influence of the phosphatase PTPN2 on their intrinsic T-cell receptor (TCR) signalling (85) and by metabolites such as glucose, tryptophan and ATP. In particular, the SYSCID map illustrates pyroptosis of pathogenic follicular helper T cells in SLE, induced by ATP-stimulated purinergic receptor P2X7 (86).

##### 3.2.2.3 Regulation of regulatory B and T cells

The main actors able to suppress the immune system are regulatory T cells (87) and regulatory B cells (88). In autoimmune diseases such as IBD, RA and SLE, multiple molecular mechanisms inhibit the suppressive function of the regulatory cells (89, 90). The SYSCID map illustrates pathways covering a range of molecular mechanisms underlying the suppression of regulatory cells function such as, for example, the activation of protein receptor TNFRSF4 (OX40) in OX40L/OX40-mediated Treg cells dysfunction (68) and the inhibition of Treg cells inhibitory function by TNF (91) and leptin (92). Additionally, three interleukins, namely IL9, IL21 and IL6, are also involved in Treg cells suppression. While IL9 negatively influences the ILC2-dependent Treg activation (93), IL21 suppresses the activation of follicular Treg cells (94–96). Regarding IL6, Svensson et al. (97) reported that this interleukin mediates the conversion of regulatory T cells into effector Th17 cells. Under IL6 influence, the fate of regulatory T cells depends on the decreased expression of the phosphatase PTPN2. When PTPN2 is expressed, the IL6-driven production of IL17A is inhibited and Treg cells keep their regulatory state. Otherwise, IL17A is produced, and T cells become Th17 cells (97).

#### 3.2.3 Mixed innate and adaptive immune system

##### 3.2.3.1 Effects of biologic drugs

Several biologic drugs used to treat IBD, RA and SLE, including abatacept, adalimumab, anifrolumab, anakinra, belimumab, canakinumab, certolizumab, etanercept, golimumab, infliximab, ixekizumab, risankizumab, rituximab, secukinumab, sarilumab, tocilizumab, vedolizumab and ustekinumab are represented in the SYSCID map, specifically in the submap “Anti-inflammatory mechanisms of selected biologics” (**Table S1**). This submap shows anti-inflammatory mechanisms of most of these biologics, excluding anakinra, canakinumab, ixekizumab, sarilumab, ustekinumab and anifrolumab, for which the map contains only direct targets. Among the biologic drugs present in the submap, vedolizumab seems to be the one that affects the greatest number of phenotypes. This drug is used to treat IBD by inhibition of the integrin α4β7 (heterodimer ITGA4-ITGB7). Through this inhibition, vedolizumab downregulates the expression of several proteins that favour disease progression and stimulates the conversion of pro-inflammatory M1 macrophages into anti-inflammatory M2 macrophage (98). Interestingly, as shown in the biologics submap, this conversion is also stimulated by adalimumab and infliximab by the mechanism of TNF receptor:Fc receptor co-stimulation. Still regarding infliximab, the submap shows this biologic restores monocytes apoptosis sensitivity via downregulation of miR-29b and consequent upregulation of the apoptosis-inducing protein HBP1, one of the miR-29b targets (99). Moreover, infliximab, as well as certolizumab pegol, favours CIDs remission through downregulation of the proinflammatory protein GDF1 via upregulation of TGFB1 in PBMCs (100). Finally, it is worth to mention that anti-TNF drugs, such as infliximab, have been demonstrated to cause clinical symptoms that mimic lupus in treated patients (101). Called anti-TNF-induced lupus (ATIL), this entity is characterized by the presence of many types of autoantibodies as represented in the map: antinuclear, anti-dsDNA, anti-histone, anti-Smith and anti-citrullinated protein antibodies (101).

### 3.3 Exploration of the SYSCID map

The SYSCID map is a graphical review of the molecular mechanisms of IBD, RA and SLE which supports integrated visualisation of omics data. Below, we describe how the map combined with genome-wide association studies (GWAS) and transcriptome data highlights potential molecular mechanisms affected by gene variants or differentially expressed genes (DEGs). Such integration is useful to detect gaps between knowledge about mechanisms of diseases and omics-derived observations. Moreover, it can lead to the discovery of potential mechanistic biomarkers, i.e., activation of cellular pathways (102) that are specific to the mechanisms underlying a given disease (14).

#### 3.3.1 Suggesting mechanistic consequences of gene variants

We collected genes harbouring variants, i.e., single nucleotide polymorphisms (SNPs), associated with IBD, RA and SLE from the GWAS catalogue (24) (see “Material and Methods”; **Tables S2** to **S5**). These genes, hereafter called disease-associated genes (DAGs), were integrated with the SYSCID map as a publicly available dataset. As a result, out of the 897 DAGs in total (**Table S2**), i) 45 matched the contents of the map for IBD (**Table S3**), ii) 53 for RA (**Table S4**), and iii) 35 for SLE (**Table S5**). For these DAGs, we performed a SYSCID map-based pathway enrichment analysis (as described in “Methods”), separately for each disease. One pathway was enriched for RA DAGs and three for IBD and SLE DAGs, as shown in **Table 1**. The single enriched pathway in RA DAGs is the “Regulation of IL10 production in Th1 cells” (adjusted p = 0.0095). Interestingly, this pathway was also enriched in IBD (adjusted p = 0.00125) and SLE DAGs (adjusted p = 0.0003) and is the top enriched pathway in all three CIDs.

**Table 1:** Annotated pathways significantly enriched (adjusted p < 0.05) in DAGs matching their corresponding proteins in SYSCID map.

Enriched pathway	Adjusted p-value		
	IBD	RA	SLE
IBD microbiota	0.0482	–	–
Regulation of IL10 production in Th1 cells	0.0125	0.0095	0.0003
Regulation of Th17 cell activity	–	–	0.0330
Regulation of the stability of gut epithelial adherens junctions	0.0125	–	–
B cell differentiation	–	–	0.0013

- Annotated pathways not significantly enriched in any DAG matching their corresponding proteins in the SYSCID map.

By integrating the GWAS catalogue data to the SYSCID map, we could indicate a specific immunological feature potentially common to these three CIDs, namely, the regulation of IL10 production in Th1 cells. The production of IL10 in IBD, RA and SLE dictates their course: while in SLE patients IL10 favours B cell differentiation and autoantibody production as shown in the SYSCID map (see pathway “IL10-mediated B cell differentiation”), a dysfunction of IL10 production makes mice prone to spontaneous colitis (103, 104). Still regarding IBD, the SYSCID map indicates that deficiency of IL10 induces chronic endoplasmic reticulum stress in the gut and promotes IBD-like disease in mice (103). Finally, in RA, unlike SLE and similar to IBD, IL10 has been linked to reduced expression of proinflammatory proteins and consequent alleviation of joint inflammation (105).

We extended the pathway enrichment analysis performed after the integration of DAGs to the SYSCID map to investigate their influence at the mechanistic level. To this end, we manually inspected the pathways of the SYSCID map for proteins encoded by the matched DAGs that directly influence other proteins.

In this regard, the pathway “Regulation of the stability of gut epithelial adherens junctions” is an interesting case. As already described in the previous section, the protein encoded by a mutated version of *INAVA* (rs41313912, missense variant, Tyr333Phe), which is associated with increased risk of IBD (54), is not able to impede the ARF6-mediated internalisation of CDH1 and the consequent destabilisation of the adherens junctions (55). This happens because this mutated version of INAVA is more prone to ubiquitination than its wild-type version. If INAVA is degraded by proteasome, then CYTH1 is stabilised and directly promotes the ARF6-mediated internalisation of CDH1 (55). Although this missense variant is not present in the GWAS catalogue as this study is not eligible for inclusion in the GWAS Catalogue (https://www.ebi.ac.uk/gwas/docs/methods), it is still an interesting example of mechanistic downstream consequences of a gene variant. Importantly, *INAVA* also has IBD-related variants according to the GWAS catalogue, namely rs12131796, rs35730213, rs55838263, rs7554511 and rs905634, and the genes *CYTH1* and *CDH1*, coding for proteins downstream to INAVA, also have IBD-related variants according to the GWAS catalogue: rs17736589 for *CYTH1*, and rs16958356 and rs16958356 for *CDH1*. Therefore, gut epithelial adherens junctions seem to be heavily affected by genetic variation.

Another example of mechanistic interpretation of GWAS data is protein encoded by a PADI2 gene bearing two intron RA-associated variants (rs2235909 and rs761426) in pathway “Induction of osteoclastogenesis by autoantibodies against citrullinated proteins”. This enzyme citrullinates other proteins. In RA, *PADI2* citrullinates, among others, the protein vimentin, a potential target of the anti–citrullinated protein auto-antibodies (ACPAs) (71). Therefore, given the intronic location of variations, the mechanistic link between *PADI2* variants and the development of RA could be an increased amount of citrullinated vimentin in synovial joint due either to a potential alternative spliced form of *PADI2* encoding for a more enzymatically active PADI2 or to an upregulation of this enzyme. There is evidence for the latter case: the RA risk T allele of rs761426 significantly increases expression levels of PADI2 in whole blood (106).

The above examples show that, through the integration of GWAS data with SYSCID map, we could identify pathways that are likely to be common to the three CIDs of interest and formulate some hypotheses about how IBD and RA can be affected by altered proteins encoded by, respectively, IBD- and RA-associated genetic variants.

#### 3.3.2 Analysis and interpretation of altered gene expression

We integrated transcriptome data with the SYSCID map to demonstrate possible mechanistic consequences related to differentially expressed genes (DEGs). To this end, we used the dataset from the study of therapy response in IBD patients treated with anti-TNF therapy, i.e., infliximab (26). We selected this study because it tries to address one of the most CIDs-related clinically relevant challenges: the low remission rates of the current treatment options for these diseases.

In this study, DEGs were calculated by comparing gene expression profiles of RNA extracted from whole blood of remitting and non-remitting IBD patients at six time points (4, 24 and 72 h and 2, 6 and 14 weeks) after infliximab exposure with gene expression profiles determined in baseline, i.e., before drug exposure. We focused specifically on DEGs determined at 4 h in remitting patients as, according to the authors, the most significant alterations occurred at this time point. Of the 2722 DEGs at 4 h (compared with baseline; **Table S6**), 79 could be matched to their corresponding proteins in the SYSCID map (**Table S7**). Among them, 29 (highlighted in **Table S7**) were considered eligible, i.e., they are modifiers of downstream molecular processes or molecules (more details in “Methods”), for the mechanistic interpretation of their alterations and possible consequences linked to the induction of remission in IBD patients after anti-TNF therapy exposure. We here selected specifically the protein TBK1 for further analysis as it is the only eligible protein that is mechanistically and directly linked to another possible altered protein, the transcription factor IRF3.

TBK1 is a serine/threonine-protein kinase involved in multiple signalling pathways, including the TLR-triggered activation of IRF3, a sequence-specific DNA binding transcription factor (dbTF) that activates the transcription of type I interferon (IFN) genes and some IFN-stimulated genes (ISG), among other target genes (47). TBK1 directly phosphorylates IRF3 and, as a result, this protein migrates to the nucleus to initiate the transcription of their target genes (47).

*IRF3* is upregulated at 4h after infliximab exposure compared to baseline only in patients that attain remission at week 14. At first sight, it is surprising to find this gene upregulated after anti-TNF therapy as we would expect an equally upregulation of IRF3’s target type I IFN genes and ISGs that, in turn, are associated with a poor clinical response to infliximab treatment (107). However, when we check which known IRF3’s target genes - according to the Dorothea gene regulatory network, https://saezlab.github.io/dorothea/ (**Table S8**) - are upregulated among the DEGs at 4h, we find no type I IFN genes and ISGs, except for *CCL5*, among the list of 14 DEGs (**Table S9**); therefore, most of the type I IFN genes and ISGs were not affected in infliximab-treated remitting patients, which is in agreement with the above-mentioned study by van Baarsen. These 14 IRF3’s target DEGs, which are not present in SYSCID map and are not currently associated with IBD, are known to be involved in antiviral defence, regulation of cell cycle transition and immune cell migration according to annotations from the Gene Ontology (GO) database (20, 21).

**Figure 4:**
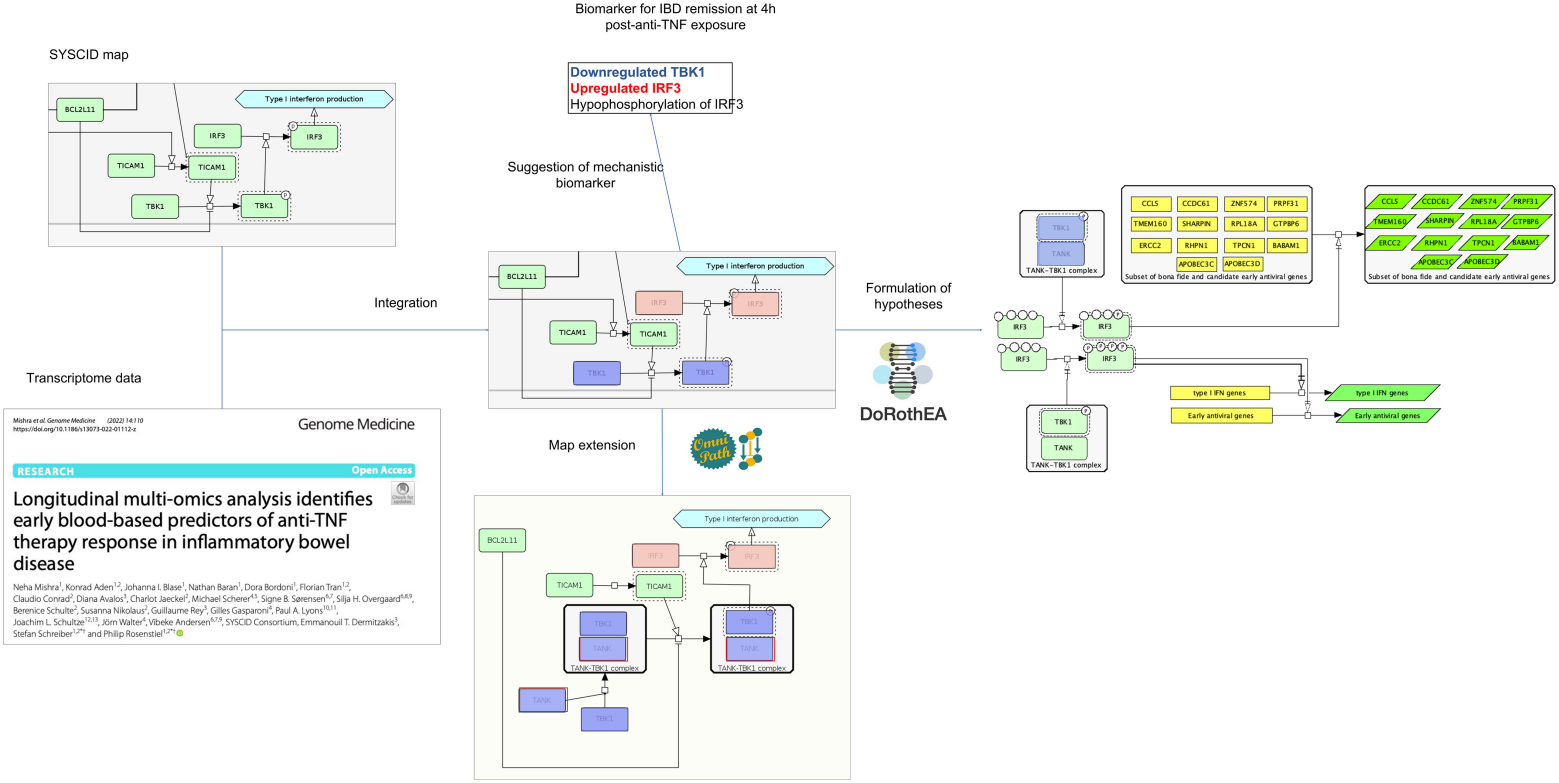
Possible outcomes after the integration of omics data within the SYSCID map Testable hypotheses can be formulated, such as that the differential transcription of IRF3 target genes is modulated by differential IRF3 phosphorylation states (right) and that TBK1 capacity to phosphorylate IRF3 is impaired by low amounts of TANK (below). TANK, in fact, was not originally present in the map; however, by checking an external signalling pathways database - in this case, Omnipath (https://omnipathdb.org/) -, this protein could be added to the map to clearly show the TANK-TBK1 interaction. Finally, potential mechanistic molecular biomarkers can be suggested; in this case, the combination of underexpressed TBK1, upregulated IRF3 and hypophosphorylated IRF3 is suggested as biomarker for remission. Light green, pink and blue-purple rectangles are, respectively, unaltered, upregulated and downregulated proteins; yellow rectangles represent genes and dark green parallelograms represent mRNAs. All proteins, mRNAs and genes are identified by HGNC symbols.

Interestingly, while *IRF3* is upregulated 4h post-exposure to infliximab, compared to baseline, *TBK1* is downregulated (**Figure 4**). As shown above, it seems that IRF3 potentially activates only the transcription of a selected set of non-type I IFN genes and non-ISGs at 4h after infliximab exposure. We therefore hypothesize that, due to lower amounts of TBK1, hypophosphorylated forms of IRF3 would be generated and such forms would be able to transcribe only these non-type I IFN genes and non-ISGs. This hypothesis relies on the experimental observation that incremental IRF3 phosphorylation progressively changes the IRF3 conformation in a way that it becomes competent for the induction of IFNB (108).

To phosphorylate and activate IRF3, TBK1 *per se* should physically interact with the scaffold protein TANK. Although this protein is not present in SYSCID map, it may also be involved in infliximab-lead remission in IBD patients since its encoding gene, *TANK*, is downregulated 4h after inflixumab exposure in comparison to baseline. So, we can hypothesize that low amounts of TANK would impair the TBK1’s capacity to phosphorylate IRF3. As TBK1 itself is also downregulated, then IRF3 phosphorylation would be greatly reduced. This fact warrants the addition of TANK to the SYSCID map and suggests that the combination of downregulated *TANK* and *TBK1* and IRF3 hypo-phosphorylation could be a potential mechanistic molecular biomarker for remission in IBD patients.

The above example shows that, through the integration of omics data within SYSCID map, we could formulate two related and testable hypotheses - (1) differential transcription of IRF3 target genes is modulated by differential IRF3 phosphorylation states and (2) TBK1 capacity to phosphorylate IRF3 is impaired by low amounts of TANK - and suggest an expansion of SYSCID map via the addition of the TANK-TBK1 interaction. Moreover, we also could suggest a potential mechanistic molecular biomarker for remission, i.e., low *TANK*, low *TBK1* and IRF3 hypo-phosphorylation (**Figure 4**). Finally, although not directed related to the content of the map but triggered by the integration of omics data within SYSCID map, we were also able to detect a new set of genes that should be investigated due to their potential involvement in the infliximab-lead remission in IBD patients.

## 4 Discussion

Representing molecular processes underlying a disease in the form of networks has become increasingly popular in systems biomedicine. Undoubtedly, the analysis and exploration of these networks have led to valuable insights into how diseases may emerge from the complex interactions between numerous biological components. Additionally, the analysis of these disease networks can also be useful for suggesting biomarkers and drug targets. However, these networks are generic, implying that interactions among their elements do not necessarily reflect true interactions under the context of a specific disease. Moreover, generic networks lack cell specificity and usually do not have proper annotations.

When building a disease map, we aim to move from the generic characterization of a disease to a more disease-specific molecular mechanistic network considering different cell types, tissues, organs and disease states (109). Based on this disease specificity, we assume that such a type of network can provide more insights into mechanisms of the disease of interest than the generic one. Although such assumption lacks formal demonstration, an increasing number of studies reporting methods to enhance generic networks specifically for a disease (110–112) indicates that scientific community has recognized how critical is a disease-specific network to better prioritize drug targets and biomarkers candidates for a disease of interest.

Based on the above-mentioned considerations and the successful applications of previous disease-specific molecular mechanistic networks, the disease maps (113–115), we opted for the construction of the network representing the IBD, RA and SLE-specific molecular mechanisms in a disease map format, i.e., the SYSCID map. We could demonstrate that this map can be useful as an interactive, graphical review of IBD, RA and SLE molecular mechanisms and, when integrated with transcriptome and GWAS data, the map can be used to indicate potential effects on altered pathways and propose possible mechanistic biomarkers.

### 4.1 Limitations of the study

It is important to emphasize that SYSCID map, as for other disease maps, does not cover all knowledge encompassing IBD, RA and SLE-specific molecular mechanisms. As this map was constructed via manual biocuration of domain experts-suggested biomedical literature on these CIDs, the molecular mechanisms present in the map reflect the coverage limitation intrinsic to the laborious and time-demanding biocuration process. Nonetheless, the SYSCID map seems to cover key mechanisms for the CIDs of interest as shown in **Figure 2** and **Table S1**. To expand the map and keep it as updated as possible, periodic revisions are planned. Finally, the long-term sustainability of the SYSCID map will be guaranteed by the ELIXIR Luxembourg’s “Disease Map” service (https://elixir-luxembourg.org/services/catalog/minerva/).

## Data availability statement

The original contributions presented in the study are included in the article/**Supplementary Material**. Further inquiries can be directed to the corresponding author.

## Author contributions

MA: Data curation, Formal Analysis, Writing – original draft, Writing – review & editing. MO: Conceptualization, Data curation, Supervision, Writing – review & editing. AM: Visualization, Writing – review & editing. PR: Funding acquisition, Project administration, Writing – review & editing. KA: Writing – review & editing. NM: Writing – review & editing. VA: Writing – review & editing. PS: Writing – review & editing. AB: Writing – review & editing. AnF: Writing – review & editing. SR: Writing – review & editing. AxF: Writing – review & editing. WG: Project administration, Writing – review & editing. RS: Project administration, Writing – review & editing. VS: Project administration, Supervision, Writing – review & editing.

## Supplementary material

Supplementary Table 1 Pathophysiological features and related pathways present in SYSCID map.

Supplementary Table 2 IBD-, RA- and SLE-associated genes extracted from GWAS Catalog on 04.11.2022.

Supplementary Table 3 IBD-associated genes extracted from GWAS Catalog on 04.11.2022 that could be integrated into the map.

Supplementary Table 4 RA-associated genes extracted from GWAS Catalog on 04.11.2022 that could be integrated into the map.

Supplementary Table 5 SLE-associated genes extracted from GWAS Catalog on 04.11.2022 that could be integrated into the map.

Supplementary Table 6 Differentially expressed genes (DEGs) from GSE191328 dataset (*t* = 4h *vs* baseline).

Supplementary Table 7 DEGs (*t* = 4h after treatment *vs* baseline) from GSE191328 dataset that could be integrated into the map.

Supplementary Table 8 IRF3 Target genes (according to Dorothea’s TF-TG network, confidence **A** to **C**).

Supplementary Table 9 IRF3 target genes (according to Dorothea’s TF-TG network; confidence **A** to **C**) in DEGs (*t* = 4h after treatment *vs* baseline) from the GSE191328 dataset.

Supplementary Table 10 Publications used for manual curation and construction of the SYSCID map.
